# Functionally active rat S100A4 from a polymerase chain reaction-synthesized gene expressed in soluble form in *Escherichia coli*

**DOI:** 10.3892/ol.2014.1870

**Published:** 2014-02-11

**Authors:** ZIQUAN LIU, CHUANXIANG XU, JIANWEI ZHANG, YUNYUN CHEN, XIAOHUA LIU, LEI WU, ZHIQING ZHANG, XIANGYAN MENG, HONGTAO LIU, ZIFENG JIANG, TIANHUI WANG

**Affiliations:** 1 Institute of Health and Environmental Medicine, Heping, Tianjin 300050, P.R. China; 2 Department of Physiology and Pathophysiology, Logistics College of Chinese People’s Armed Police Force, Hedong, Tianjin 300162, P.R. China; 3 Tianjin University of Sport, Nankai, Tianjin 300381, P.R. China; 4 Institute of Zoology, Chinese Academy of Sciences, Chaoyang, Beijing 100101, P.R. China

**Keywords:** rat S100A4, functional expression, gene recombination

## Abstract

S100A4 protein is associated with Ca^2+^-dependent regulation of intracellular activities and is significant in the invasion, growth and metastasis of cancer. In order to express rat S100A4 functionally and identify its biological activity following purification, an S100A4 gene fragment was optimized and fully synthesized via overlapping polymerase chain reaction. The gene was inserted into the prokaryotic expression vector, pBV220, with phage λ P_R_P_L_ promoters following confirmation by DNA sequencing. The pBV220-S100A4 plasmid was constructed and transformed into *Escherichia coli* DH5α. Following temperature induction, rat S100A4 was overexpressed and the protein was observed to be located in the supernatant of the lysates, which was ~30–40% of the total protein within the host. The protein was isolated and purified by metal-chelate affinity chromatography. High purity protein (>98% purity) was obtained and *in vitro* western blot analysis identified that the recombinant S100A4 was able to bind to the antibody against wild-type S100A4. The bioactivity of the recombinant protein was detected via Transwell migration and invasion assays. The polyclonal antibody of rat S100A4 protein was prepared for rabbit immunization and exhibited similar efficacies when compared with commercial S100A4. Therefore, rat S100A4 was functionally expressed in *E. coli*; thus, the production of active recombinant S100A4 protein in *E. coli* may further aid with the investigation and application of S100A4.

## Introduction

S100A4 protein, a polypeptide containing 101 amino acids with a molecular weight of 11.5 kDa, is associated with Ca^2+^-dependent regulation of intra- and extracellular activities, such as protein phosphorylation, enzyme activity and cell motility (1–4). Previous studies into the roles of S100A4 have predominantly focused on the invasive growth and metastasis of cancer (5–7). The increased expression of S100A4 is correlated with breast, colorectal and gastric carcinoma, and it has been suggested that S100A4 may be used in the early diagnosis and prognosis of cancer as a complementary specific biomarker (8,9). However, problems such as preparation complexity and cross-reactivity of available antibodies impede further investigation into the biochemical roles and distribution of the S100A4 protein. Therefore, the possibility of generating whole S100A4 via recombinant techniques may be advantageous in such applications. In the present study, the construction and expression of a synthetic gene that encodes S100A4 within *Escherichia coli*, is described. The expressed S100A4 was highly soluble and stable, as well as functionally active. Thus, the present study provides a foundation for further investigation and for application of S100A4 in clinical studies.

## Materials and methods

### Reagents

Cloning vector pEasy-T3, vector pBV-220 and *E. coli* DH5α were preserved in the Performance Medicine Laboratory of the Institute of Health and Environmental Medicine (Tianjin, China). A gel extraction kit, plasmid extraction kit, *Bam*HI, *Eco*RI and T4 DNA ligase were purchased from Promega Corporation (Madison, WI, USA). Taq DNA polymerase and Pfu DNA polymerase were obtained from Tiangen Biotech (Beijing, China). Rat S100A4 multifunctional antibody was purchased from Santa Cruz Biotechnology, Inc. (Santa Cruz, CA, USA). Acrylamide and N, N′-methylenebisacrylamide were purchased from GE Healthcare Life Sciences (Pittsburgh, PA, USA). SDS, 3,3′-diaminobenzidine tetrahydrochloride and staphylococcal protein A (SPA) sepharose CL-4b column chromatography equipment was procured from Sigma (St. Louis, MO, USA). All other chemicals were obtained from Shanghai Sangon Biological Engineering Technology and Service Co., Ltd. (Shanghai, China). Cell culture inserts were purchased from Millipore (Billerica, MA, USA) and 24-well cell culture plates were acquired from Corning Co., Ltd. (Corning, NY, USA). Matrigel was purchased from BD Biosciences (Franklin Lakes, NJ, USA) and complete and incomplete Freund’s adjuvant was obtained from Beijing Dingguo Biotechnology Co., Ltd. (Beijing, China). The remaining chemical reagents used in the experiments were of analytical grade. This study was approved by the ethics committee of the Institute of Health and Environmental Medicine (Tianjin, China).

### Construction of cloning vector pEasy-T3 S100A4

The gene fragment of rat S100A4 was constructed by overlapping polymerase chain reaction (PCR) using 12 synthetic oligonucleotides as primers (Table I). The primers were synthesized based on the codon preference within *E. coli*. The forward primer, pbf104; 5′-GCTGAATTCATGGCGCGTCCGCTGGAAG-3′ contained a site for *Eco*RI (underlined), whereas the reverse primer, pbr104; 5′-GCA**GGATCC**TTAATGATGGTGGTG ATGATGC-3′ contained a site for *Bam*HI (bold sequence), 6X His tags and a stop codon (underlined). Standard PCR was used to amplify the full-length coding sequence using the designed primers as templates. The primer concentrations decreased from each end towards the middle: Primers 1 and 12, with a concentration of 20 nmol/l; primers 2 and 11, with a concentration of 10 nmol/l; primers 3 adn 10 with a concentration of 5 nmol/l; primers 4 and 9 with a concentration of 2..5 nmol/l; primers 5 and 8 with a concentration of 1.25 nmol/l and primers 6 and 7, with a minimum concentration of 0.625 nmol/l. PCR amplification was initially performed over five cycles of predenaturation at 94°C for 3 min, denaturation at 94°C for 30 sec, annealing at 45°C for 30 sec and extension at 72°C for 30 sec, followed by a further 25 cycles of denaturation at 94°C for 30 sec, annealing at 55°C for 30 sec and extension at 72°C for 30 sec. The products were digested with the corresponding restriction enzymes and ligated into the pEasy-T3 vector. S100A4-pEasy plasmid was sequenced to verify the integrity of S100A4 (Invitrogen Life Technologies, Carlsbad, CA, USA).

### Expression of rat recombinant S100A4 protein

The coding region of rat S100A4 in the cloning vector was digested by *Eco*RI and *Bam*HI and was ligated into the PBV220 vector. The pBV220-S100A4 plasmid was transferred into *E. coli* DH5α. A single bacterial colony was inoculated in 5 ml Luria-Bertani (LB) media containing 50 μg/ml ampicillin and was placed on a rotary shaker at 30°C overnight; the seed cultures were subsequently transferred to 500 ml LB media containing ampicillin. As the optical density (OD)-600 of the culture reached 0.8, induction was initiated by heating; the culture was induced for 4 h at 42°C and collected by centrifugation at 5000 × g for 10 min. Thereafter, the culture was suspended in 1X phosphate-buffered saline (PBS) containing 10 mM mercaptoethanol and 1 mM phenylmethylsulfonyl fluoride. The culture was sonicated in an ice bath, the lysate was centrifuged at 5000 × g for 15 min, and the supernatant was determined by SDS-PAGE and stained with Coomassie Brilliant Blue, to confirm the S100A4 expression. The fusion protein quantity was assessed through comparison with the bands formed by the standard protein.

### Purification of rat recombinant S100A4

Metal-chelate affinity chromatography (5 ml HiTrap HP; GE Healthcare Life Sciences) was used to purify the rat recombinant S100A4 using the Amersham fast-protein liquid chromatography (FPLC) purification system (Harlow Scientific, Arlington, MA, USA). The supernatant was diluted five times with 1X binding buffer (containing 0.1 mol/l guanidinium hydrochloride) prior to filtering and the generated liquids were discarded after the column was balanced to the baseline, according to the manufacturer’s instructions. Step gradient elution was subsequently conducted with the elution buffer and the recombinant proteins were collected and confirmed via SDS-PAGE.

### Western blot analysis

The purified protein was transferred to a nitrocellulose membrane following SDS-PAGE, using a semi-dry electrophoretic transfer device (Jim-X Biotechnology Co., Ltd., Dalian, China). The membrane was blocked with 3% bovine serum albumin (BSA) in PBS containing 0.5% Tween-20 and was incubated with rabbit polyclonal antibody against rat S100A4 (Santa Cruz Biotechnology, Inc.) and horseradish peroxidase (HRP)-coupled goat anti-rabbit IgG secondary antibody (Abcam (Hong Kong) Ltd., Hong Kong, China)

### Protein assay

The protein concentrations in the samples were determined using a Bradford protein assay kit (Sangon Biotech, Shanghai Co., Ltd, Shanghai, China) with BSA at the standard concentration (10).

### SDS-PAGE analysis

SDS-PAGE analysis was performed under denaturing conditions using the method described by Laemmli (11). The concentrations of the stacking and resolving gels were 5 and 15%, respectively.

### Bioactivity of the recombinant protein

Recombinant protein bioactivity was identified by Transwell migration and invasion assays (12). Transwell invasion chambers were coated with Matrigel and the assays were conducted according to the manufacturer’s instructions. HeLa cells (1×10^5^) with 50 μg/ml recombinant S100A4 protein were placed in the top chambers and served as the experimental group, while HeLa cells (1×10^5^) without S100A4 protein were used as the control group. The cells were incubated for 24 h at 37°C and the motile cells at the top of each chamber were removed with cotton swabs. The cells at the bottom of each chamber were fixed with 0.1% glutaraldehyde for 30 min, rinsed briefly with PBS and stained with 0.2% crystal violet for 20 min. The chambers were washed thoroughly with PBS and the number of migrating cells or invasive cells was tallied using ×200 magnification (Olympus CKX31, Olympus, Tokyo, Japan); the mean number of cells per chamber was also determined. The results were calculated as the migration/invasion rate relative to the parental control cells. Each experimental condition was duplicated and repeated three times.

### Polyclonal antibody preparation

Antibodies against rat S100A4 protein were raised within New Zealand white rabbits obtained from the Experimental Animal Center of the Institiue of Health and Environmental Medicine Research (Heping, China). The immunization procedure was implemented as follows: On day one, 200 μg purified antigen was mixed with an equal volume of complete Freund’s adjuvant, which was multipoint injected subcutaneously into the back of each rabbit. The rabbits were then boosted subcutaneously four times with 200 μg recombinant protein in incomplete Freund’s adjuvant at 10 day intervals. The antiserum was collected and determined 10 days subsequent to the last injection. Purification of rabbit IgG was performed according to the methods described by Moro *et al* (13) with minor modifications. The IgG fraction was purified by precipitation with 100% saturated (NH_4_)_2_SO_4_ and by passing the IgG fraction through the SPA-sepharose CL-4b column chromatograph.

### Antibody titer determination

Antiserum titers were examined using indirect ELISA. The wells of the polystyrene microtiter plates were coated with an antigen and, following overnight incubation at 4°C, the coated microtiter plates were blocked with BSA. The wells were incubated for 15 min in the dark at room temperature, with polyclonal antibodies against S100A4 with different deliquations (from 1:100 to 1:100,000) and HRP-conjugated goat anti-rabbit IgG (dilution, 1:3,000); the peroxidase substrate was tetramethyl benzidine. Following incubation, the reaction was stopped using 2 M H_2_SO_4_ and the absorbance at was measured at an OD of 450 nm, using a microplate reader (Multiskan MK3; Thermoscientific, Waltham, MA, USA).

### Statistical analysis

All results are expressed as the means ± SD. Data were analyzed using SPSS version 17 (SPSS, Chicago, IL, USA). P<0.05 was considered to indicate a statistically significant difference.

## Results

### Design, cloning and analysis of the S100A4 synthetic gene

**Figure 1:**
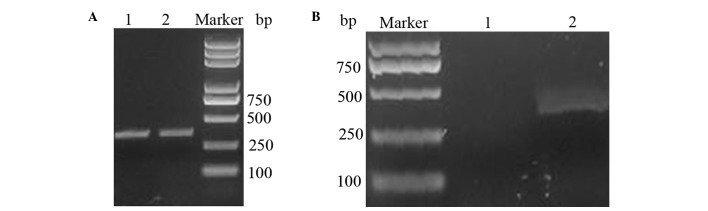
(A) Agarose gel analysis of S100A4 DNA. Lanes 1 and 2, S100A4 DNA; Marker, DNA marker. (B) Agarose gel analysis of pBV220-S100A4 vector following digestion by *Bam*HI and *Eco*RI. Lane 1, pBV220-S100A4 plasmid; lane 2, pBV220-S100A4 digested by *Bam*HI and *Eco*RI; Marker, DNA marker.

The natural gene sequence (GenBank accession no. NM012618) was used as the template for designing and optimizing S100A4 according to the *E. coli* codon usage. Thirty-four rare codons of the natural gene were replaced with synonymous high-frequency codons in a synthetic gene that encodes S100A4. A 300-bp fragment was synthesized using three-step PCR (Fig. 1A). The PCR-synthesized sequence cloned into the pEasy-T3 vector was digested by *Eco*RI and *Bam*HI, thereby producing the expected band size.

### Construction of the expression vector, pBV220-S100A4

The pEasy-S100A4 plasmid was sequenced to verify the integrity of S100A4. Following confirmation by DNA sequencing and *Eco*RI and *Bam*HI digestion, the S100A4 gene was successfully cloned into the expression vector, pBV220-S100A4. Agarose gel analysis of the digestion of pBV220 is shown in Fig. 1B.

### Recombinant S100A4 protein expression

**Figure 2:**
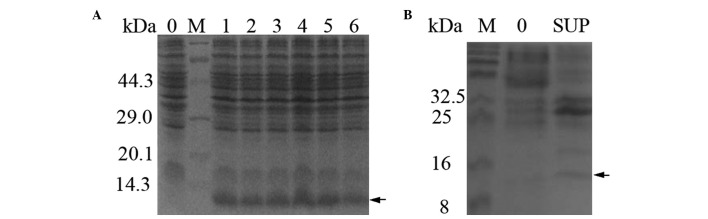
Expression of recombinant S100A4. (A) SDS-PAGE analysis of bacterial cultures grown in Luria-Bertani broth medium showed increasing expression of the recombinant protein (arrow) induced by heat shock. (B) SDS-PAGE analysis of bacterial cultures on the supernatant fraction. Lane 0, negative control; lane M, protein standards; lanes 1–6, induced products of pBV220-S100A4; lane SUP, induced products of pBV220-S100A4 in the supernatant fraction.

The pBV220-S100A4 plasmid was transferred into *E. coli* DH5α and was induced using heat shock. The 11.5 kDa S100A4 fusion protein was expressed at a high level, ~20–30% of the total protein of the cell, as analyzed by Total Lab 2.01 software (Newcastle, UK; Fig. 2A). The expected 11.5 kDa band was apparent in the supernatant and absent in the negative control (Lane 0; Fig. 2B). The SDS-PAGE analysis indicated that the S100A4 protein was expressed in a soluble form.

### Recombinant S100A4 purification

**Figure 3:**
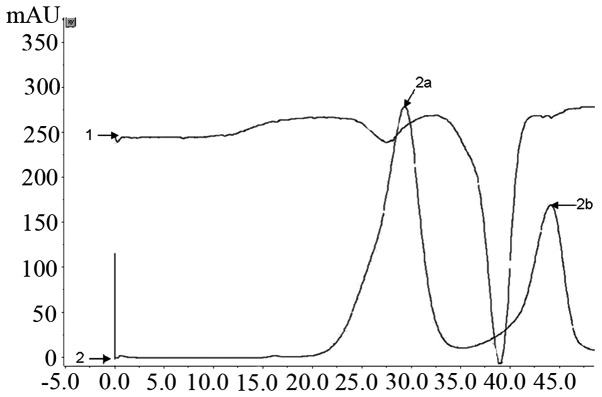
Chromatogram of the purification process. Line 1, electric conductance; line 2, eluted protein; peak 2a, proteins excluding S100A4; peak 2b, S100A4 protein.

**Figure 4:**
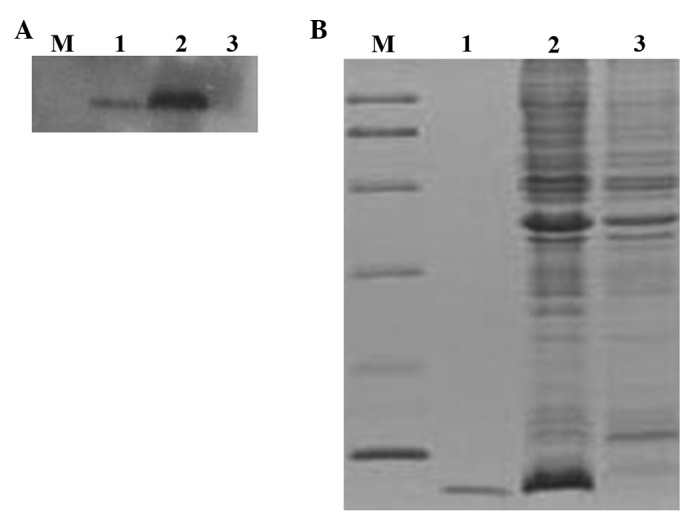
Identification of recombinant S100A4. (A) Western blot analysis of the recombinant protein. (B) SDS-PAGE analysis of the recombinant protein. Lane M, protein standards; lane 1, purified S100A4 protein; lane 2, induced products of pBV220-S100A4; lane 3, negative control.

The 6X His-tagged S100A4 protein was one-step purified by the Ni^2+^ affinity chromatography column using the FPLC purification system. The column was washed with 200 mmol/l imidazole gradient elution buffer at 3 ml/min and ~15 ml concentrated eluted fluid was obtained (Fig. 3). The SDS-PAGE analysis identified that the protein remained intact and the purity of the final S100A4 was 95% following desalination with HiTrap Desalting (GE Healthcare Life Sciences; Fig. 4B). The yielded purified recombinant protein was ~50 mg/l of the induced culture.

### Western blot analysis of recombinant S100A4

The rabbit polyclonal antibody was capable of recognizing natural S100A4 and recombinant S100A4 protein, and the western blot analysis identified that the recombinant S100A4 was functionally expressed (Fig. 4A).

### Migration and invasion of HeLa cells

**Figure 5:**
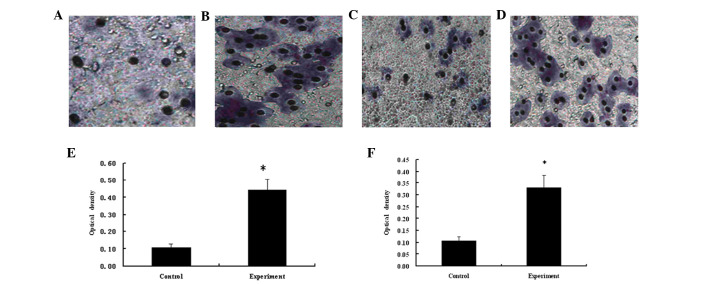
Increased invasion and migration of HeLa cells by the recombinant protein. (A) HeLa cells (1×10^5^) without recombinant S100A4 protein in the Transwell migration assay. (B) HeLa cells (1×10^5^) with recombinant S100A4 protein in the Transwell migration assay. (C) HeLa cells (1×10^5^) without recombinant S100A4 protein in the Transwell invasion assay. (D) HeLa cells (1×10^5^) with recombinant S100A4 protein in the Transwell invasion assay. (E) OD of HeLa cells in the migration experiment. (F) OD of HeLa cells in the invasive experiment. OD, optical density. ^*^ P<0.05, compared with the control group.

The Transwell migration and invasion assays identified that the number of cells in the experimental group was greater than that of the control group (Fig. 5A–D), thus identifying that the recombinant protein was capable of improving the motility and invasiveness of cancer cells. A four-fold increase was observed in the number of migrating cells in the experimental group compared with the control group (P<0.05; Fig. 5E). The invasive ability of the HeLa cells in the experimental group was determined using the Matrigel invasion assay, which identified that the invasive ability of HeLa cells in the experimental group was three-fold greater than that of the control group (P<0.05; Fig. 5F). This therefore indicated that the recombinant protein levels promoted the motility and invasiveness of cancer cells.

### Titer and specificity of polyclonal antibody

**Figure 6:**
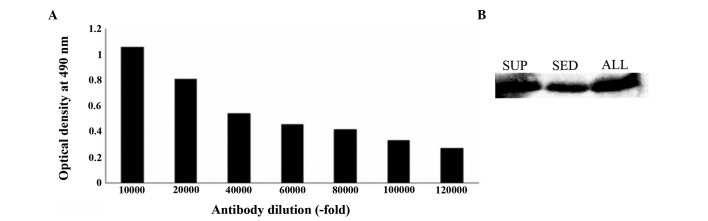
Analysis of anti-S100A4 antiserum. (A) Analysis of anti-S100A4 titer. The antibody titer was measured using ELISA. The antibody at varying dilutions (10,000–120,000 fold) or reactions with equal quantities of purified protein (1 mg/ml). Antibody titer was defined as the dilution of the antibody corresponding to the antibody dilution of 0.500 at an optical density of 490 nm. (B) Western blot analysis of rabbit polyclonal antibody against the recombinant protein. SUP, induced products of pBV220-S100A4 in the supernatant; SED, induced products of pBV220-S100A4 in the precipitate fraction; ALL, induced products of pBV220-S100A4 in all fractions.

Following rabbit immunization with recombinant S100A4, the antiserum was purified by protein G affinity chromatography. The anti-S100A4 antibody concentration was 0.78 mg/ml and the antibody titer, determined using ELISA, was ~1:120,000 (Fig. 6A). The antiserum obtained was specific to the recombinant protein according to the western blot analysis (Fig. 6B).

## Discussion

In the present study, a pBV220-S100A4 vector was successfully constructed based on an optimized S100A4 gene. The soluble S100A4 protein was expressed at an increased level following transferal of the constructed expression vector into *E. coli* DH5α and induction via heat shock. The purity of the S100A4 protein was improved following purification via revised Ni affinity chromatography. Additional proteins with similar structures that belong to the same protein family were also obtained, such as S100A1 and S100B, using the same methodology. The present study identified that the expression system was efficient and stable (15).

It has been shown that the synthesis of a full-length gene may contribute to the expression DH5α in *E. coli* by the optimized gene (16,17). There was an initial attempt to synthesize the S100A4 gene by one-step PCR; however, following several cloning procedures, it was not continued as one or two frameshift or lethal mutations occurred in the synthesized sequence. These mutations may have been a result of the mismatch between overlapping primers. Therefore, two short fragments were synthesized by PCR and subsequently used to obtain the full-length gene by performing an additional PCR. This process was conducted to reduce the mismatch by decreasing the quantity of overlapping primers, which was reasonable and aided synthesis of long DNA fragments via PCR.

Studies have shown that pET30a(+) was used as the expression vector of the S100A4 protein (17,18). However we used plasmid pBV220 as the expression vector in our experiment. The results of the present study demonstrated that the soluble S100A4 protein was expressed at a high level and accounted for >20% of the total protein levels, which was greater than that observed in pET30a(+). Plasmid pBV220, which was constructed at the National Institute for Viral Disease Control and Prevention, is an efficient cloning and expression vector that is regularly used in China (Beijing, China) (14). The temperature, induced by P_R_P_L_ double promoters, controls the pBV220 plasmid. The gene was inserted into pBV220 and was highly expressed in *E. coli* DH5α, which exhibits rapid growth and reproduction, low nutrition requirements and is readily manipulated. *E. coli* DH5α is an optimal expression system for proteins that are capable of biological activity *in vitro*. The present study identified that soluble S100A4 protein was expressed at a high level and accounted for >20% of the total protein.

The present study also aimed to obtain increasingly purified S100A4 proteins. In the initial purification investigation, soluble S100A4 was not successfully combined with certain metal ions and the penetrating fluids contained numerous target proteins. This may be due to the inability of the 6X His tag to be fully exposed during the protein folding process. Therefore, the common Ni affinity chromatography method was revised and following the addition of 0.1 mol/l guanidine hydrochloride into the binding buffers, the binding capacity was significantly improved and the protein was further purified. Thus suggesting that guanidine hydrochloride, or other denaturing agents at an appropriate concentration, are capable of effectively improving the binding as a result of increased exposure of the His tag to the target protein.

The Transwell migration and invasion assays showed that the recombinant protein levels promoted the motility and invasiveness of the cancer cells. Antiserum was obtained from immunized rabbits using S100A4 as the immunogen; the polyclonal antibody obtained was identified as possessing highly specific and sensitive affinities to the protein.

As anti-S100A4 antiserum was able to bind as strongly to recombinant S100A4 as to natural S100A4, the basic biological activity of recombinant S100A4 was retained. Thus, the expression vector, pBV220-S100A4, was successfully constructed based on the 6X His-tagged N-terminal S100A4 gene. The 6X His-tagged recombinant pBV220-S100A4 protein was purified using revised Ni affinity chromatography. In conclusion, the production of the active recombinant S100A4 protein within *E. coli* may aid with further investigation and applications of S100A4.

## Figures and Tables

**Table I:** Oligonucleotide primers with mutual overlaps.

Primer	Primer sequence	Length of primers (bp)
1	GCTCCATGGCGCGTCCGCTGGAAGAAGCGCTGGATGTGATTGT	43
2	CATTGCCGCTATACTTATGAAAGGTGCTCACAATCACATCCAGCGCTTCT	50
3	ACCTTTCATAAGTATAGCGGCAATGAGGGCGACAAATTCAAACTGAATAA	50
4	GGGTCAGCAGTTCTTTCAGTTCGGTTTTATTCAGTTTGAATTTGTCGCCC	50
5	GAACTGAAAGAACTGCTGACCCGTGAACTGCCGAGCTTTCTGGGCCGTCG	50
6	TTCATCAGTTTCTGAAACGCCGCTTCATCGGTACGACGGCCCAGAAAGCT	50
7	CGGCGTTTCAGAAACTGATGAATAATCTGGATAGCAATCGTGATAATGAA	50
8	ACACGCAATATTCCTGAAAATCCACTTCATTATCACGATTGCTATCCAGA	50
9	TGGATTTTCAGGAATATTGCGTGTTTCTTAGCTGCATTGCGATGATGTGC	50
10	TTTATCCGGGCAGCCTTCGAAAAATTCATTGCACATCATCGCAATGCAGC	50
11	CGAAGGCTGCCCGGATAAAGAACCGCGTAAAAAGCATCATCACCACCATC	50
12	GCCAAGCTTTTAATGATGGTGGTGATGATGCTTTTTAC	38
pbf104	GCT**GAATTC**ATGGCGCGTCCGCTGGAAG	
pbr104	GCA**GGATCC**TTAATGATGGTGGTGATGATGC	

pbf104, forward; bold print denotes a site for EcoRI. pbr104, reverse; bold print denotes a site for BamHI. Underlining denotes a stop codon.
